# RBCK1 promotes p53 degradation via ubiquitination in renal cell carcinoma

**DOI:** 10.1038/s41419-019-1488-2

**Published:** 2019-03-15

**Authors:** Sifan Yu, Jie Dai, Meng Ma, Tianxiao Xu, Yan Kong, Chuanliang Cui, Zhihong Chi, Lu Si, Huan Tang, Lu Yang, Xinan Sheng, Jun Guo

**Affiliations:** Key Laboratory of Carcinogenesis and Translational Research (Ministry of Education/Beijing), Department of Renal Cancer and Melanoma, Peking University Cancer Hospital & Institute, Beijing, China

## Abstract

Renal cell carcinoma (RCC) accounts for approximately 3% of adult malignancies, and the incidence of RCC continues to rise worldwide. Although RCC can be treated with surgery at an early stages, the five-year survival rates have been observed to decline dramatically in patients with advanced disease. Most patients with RCC treated with cytotoxic or targeted drugs will develop resistance at some point during therapy. Thus, it is necessary to identify novel therapeutic targets for RCC. Here, we found that RANBP2-type and C3HC4-type zinc finger-containing 1 (RBCK1) expression was upregulated in human RCC samples. Analysis of multiple public databases revealed the correlation between RBCK1 expression and poor prognosis in RCC patients. Subsequently, we performed RBCK1 depletion experiments in RCC cells that severely affected the in vivo and in vitro proliferation of renal cancer cells. The effects of RBCK1 on cell proliferation could be rescued with p53 expression knockdown in two cell lines expressing wild-type p53. Further experiments demonstrated that RBCK1 could facilitate p53 poly-ubiquitination and degradation by direct interaction with p53. Together, our results show that RBCK1 may serve as a promising target for RCC therapy by restoring p53 functions.

## Introduction

Renal cell carcinoma (RCC) represents 2 to 3% of all cancers and is the tenth most common cancer worldwide^1,2^. Major RCC subtypes include clear cell RCC (ccRCC), papillary RCC, chromophobe RCC, collecting duct RCC and unclassified RCC^3^. ccRCC is the most common subtype accounting for 75–80% of all the RCC cases^4^. Approximately 20% of patients with RCC present with advanced stage disease at the time of diagnosis, and nearly 30% of patients with localized RCC will develop recurrence and metastasis after tumor resection^5^. Advanced RCC is a lethal disease portending a 5-year survival of only 11.7%^6^. For advanced metastatic disease, systemic treatment comprising inhibition of vascular endothelial growth factor (VEGF) pathways is available. Several tyrosine-kinase inhibitors have been recently developed tto target VEGF signaling in ccRCC and have shown significantly improved outcomes for metastatic RCC patients^7^. Sunitinib (Sutent) and pazopanib (Votrient) were approved for the first-line treatment of metastatic RCC^8^, whereas axitinib (Inlyta) and sorafenib (Nexavar) are used as second-line therapy to improve the progression-free survival^9^. However, drug resistance typically develops within 6–12 months^10^. Moreover, a significant group of patients (circa 1/4) failed to respond to the targeted first-line treatment^11^. Therefore, it is critical to further characterize the signaling pathways underlying RCC with the eventual aim to identify novel therapeutic strategies.

RANBP2-type and C3HC4-type zinc finger-containing 1 (RBCK1, also known as HOIL-1L) is a 58 kDa protein comprising an N-terminal ubiquitin like (UBL) domain, an Npl4-type zinc finger (NZF) domain and a catalytic C-terminal RBR domain^12^. Many E3 ubiquitin ligases are known to exhibit abnormal expresseion in tumors, making them valuable diagnostic markers and drug targets^13^. Previous studies have revealed that RBCK1 mRNA level was higher in breast cancer samples as compared with adjacent non-tumor tissues, and the downregulation of RBCK1 was associated with reduced level of estrogen receptor alpha and slow proliferation of breast cancer cells.Thus, RBCK1 may regulate cell cycle progression and proliferation by supporting the transcription of estrogen receptor alpha^14,15^. In patients with lung cancer, the high expression of RBCK1 was thought to be associated with adaptive hypoxia^16^. However, the expression and biological function of RBCK1 in RCC are still unknown.

In the present study, we performed RNA sequencing (RNA-seq) in RCC cells after RBCK1 depletion. RNA-seq data revealed that RBCK1 could serve as a novel regulator of p53 in RCC cells. The tumor suppressor protein p53 as a “guardian of the genome” was discovered 30 years ago and is known for its crucial role in coordinating cellular responses to genotoxic stress^17,18^. However, recent studies have shown that p53 plays multiple regulatory functions in diverse biological processes such as autophagy, metabolism, and aging^19^. p53 is frequently observed with a loss of function and induction of cell cycle arrest and apoptosis^20^. According to previous results, p53 has a low mutation rate in renal cancer (about 2–3%)^21,22^. We hypothesized that the ubiquitin protein RBCK1 could serve as an oncogene of RCC. The mechanism underlying the inhibitory effects of RBCK1 on cell proliferation may be related to the regulation of p53 ubiquitination and promotion of p53 degradation, leading to the suppression of p53 target genes. Our research aims to investigate the role of the ubiquitin protein RBCK1 in RCC and its relationship with p53. We hypothesize a novel regulatory role of RBCK1 involving p53 that may deem RBCK1 as a new therapeutic target for RCC.

## Materials and methods

### Cell culture

Two human RCC cell lines (Caki-1 and 769-P, both expressing wild-type p53) and HEK293 cells were perchased from Cell Resource Center, Institute of Basic Medical Sciences, CAMS/PUMC (which is the headquarter of China Infrastructure of Cell Line Resource, National Sciences and Technology Infrastructure, NSTI). All cell lines were cultured in Dulbecco’s modified Eagle’s medium supplemented with 10% fetal bovine serum (FBS both from Gibco Thermo Fisher Scientific) at 37 °C in a 5% CO2 humidified incubator. Cisplatin was provided by Peking Cancer Hospital.

### Small-interfering RNA (siRNA) transfection and plasmids information

The siRNAs targeting RBCK1 were designed and synthesized by RiboBio (Guangzhou, China). The sequences are listed in Table S1. A non-targeting siRNA (siControl) was used as a negative control. Cells were transfected using Lipofectamine RNAi-MAX (Invitrogen, Shanghai, China) with 10 nmol/L siRNA.FLAG-Pcdna3.1EGFP-RBCK1, mutants (ΔUBL, ΔNZF, ΔRBR-C), or Pcdna3.1 control expression plasmids were purchased from HANBIO Biological (Shanghai, China). Pcdna3.1EGFP-p53 full length and mutants were provided as gifts from Professor Jian Zhu (Henan Collaborative Innovation Center of Molecular Diagnosis and Laboratory Medicine). Transient transfection of plasmids into renal cancer cells was performed using Lipofectamine 3000 (Invitrogen, Shanghai, China) following the manufacturer’s protocol. For stable transfection, a lentiviral vector silencing RBCK1 expression was designed and synthesized by HANBIO (Shanghai, China). Lentiviruses were mixed with Polybrene (10 μg/mL) and added to renal cancer cells. Positive clones were selected with puromycin (5 mg/mL). Stable transfectants were isolated after 2 weeks.

### Western blotting analysis

Cells were lysed with radioimmunoprecipitation assay lysis buffer and the lysates containing 50 μg of total protein were subjected to sodium dodecyl sulfate polyacrylamide gel electrophoresis (SDS-PAGE; Beyotime, China). The separated protein bands were transferred onto polyvinylidene fluoride membranes (Millipore, Billerica, MA, USA). The membranes were incubated with primary antibodies overnight at 4 °C, then washed thrice with TBST buffer for 10 min each and incubated with a horseradish peroxidase-conjugated secondary antibody at room temperature (23 °C) for 1 h. Chemiluminescent HRP substrate (Millipore, Billerica, MA, USA) was added to visualize the protein bands. Anti-p53 (Do–1, AB1101), anti-RBCK1 (ab38540), anti-α-Tubulin (ab52866) and anti-histone-3(ab176840) were purchased from Abcam (Cambridge, MA, USA). While while anti-hemagglutinin (HA) (clone16B12) was supplied by Covance (Chantilly,VA,USA). For interaction experiment, anti-RBCK1 (26367–1-AP) was obtained from Proteintech (IL, USA). Anti-FLAG OctA-Probe (H-5) (sc-166355) was acquired from Santa Cruz Biotechnology (Dallas, Texas, USA), while anti-glyceraldehyde 3-phosphte dehydrogenase (GAPDH) (14C10) was supplied by Cell Signaling Technology (Danvers, MA, USA).

### RNA extraction and quantitative polymerase chain reaction (PCR) analysis

Total RNA was extracted by lysing cells with a Qiagen kit and total RNA was converted to first-strand cDNA using the High Capacity cDNA Reverse Transcription Kit (Thermo Fisher Scientific). RT-PCR was performed using the Applied Biosystems 7500 Fast Real-Time PCR System and SYBR Green, according to the manufacturer’s instructions (Thermo Fisher Scientific). 36B4 was used as an internal control. Primer sequences are shown in Table S1. The 2^−△△CT^ method was used to determine relative gene expression levels. Each experiment was repeated at least thrice.

### Mitochondrial membrane potential (MMP) analysis

Alterations in MMP were analyzed with flow cytometry using the MMP assay kit with JC-1, a marker of mitochondrial activity (Beyotime Company, Hangzhou, China). Mitochondrion from a normal undamaged nucleate cell exhibits high MMP. The breakdown of MMP is often linked to early apoptosis. JC-1 is the most widely used dye for the detection of mitochondrial depolarization during the early stages of apoptosis^23^. Caki-1 cells were transfected with 50 nM siRBCK1 or control. After 24 h, flow cytometry was performed to evaluate the MMP. JC-1 exhibits a potential-dependent accumulation in mitochondria that is indicated by a fluorescence emission shift from green to red. Cells containing J-aggregates exhibit high MMP and show red fluorescence (590 nm, FL-2 channel), while those with low MMP have the monomeric form of JC-1 that exhibits green fluorescence (530 nm, FL-1 channel). After different treatments with siRNA for 24 h, Caki-1 cells were collected and incubated with 0.5 mL of JC-1 working solution for 20 min at 37 °C. The cells were washed, resuspended in medium, and analyzed by flow cytometry.

### Flow cytometry

Propidium iodide (PI) is a fluorescent dye that stains DNA and RNA and is used to analyze cell cycle by flow cytometry. Caki-1 or 769-P cells were seeded at a density of 10^5^ cells/well in 6 cm dishes. After 24 h, the cells were transfected with corresponding siRNA or siControl. The cells were incubated for 24 h and fixed with 70% ethanol overnight at 4 °C, followed by staining with PI (BD Biosciences, 550825).

### Cell counting kit (CCK)-8 assay

The transfected cells were seeded into 96-well plates at a density of 2 × 10^3^ cells/well and incubated at 37 °C in a 5% CO_2_ humidified incubator. CCK-8 assay was conducted to detect cell proliferation at 0, 24, 48, and 72 h after inoculation. In brief, 10 µL of CCK-8 reagent (Dojindo Molecular Technologies, Shanghai, China) was added to each well at the indicated time points, and the transfected cells were incubated at 37 °C for an additional 2 h. The optical density at 490 nm wavelength was determined. Each experiment was performed in sextuplicates.

### In vivo tumorigenicity assay

All animal experiments were carried out in accordance with the NIH Guide for the Care and Use of Laboratory Animals with protocols approved by the Animal Care and Use Committee at Peking University Cancer Hospital & Institute. For the tumorigenicity assay, Caki-1-RBCK1 Control and Caki-1 short-hairpin (sh)-RBCK1 cell suspensions (5 × 10^6^ cells/mouse) in solution were injected into 6-week-old NOD-SCID mice. Twelve mice injected with each cell line were divided into 2 groups as follows: mice injected with control cells (*n* = 6) and mice injected with shRBCK1 cells (*n* = 6). In the rescue experiments, 16 mice were divided into four groups (*n* = 4). sh-Control, sh-RBCK1, sh-p53 and sh-RBCK1 + sh-p53 cell suspensions (5 × 10^6^ cells/mouse) in solution were injected into 6-week-old NOD-SCID mice. Tumor growth was monitored every 2–3 days and the tumor volumes were calculated by the formula, length × width^2^/2. The mice were sacrificed at 50 days after cell transplantation.

### RNA-seq analysis

Caki-1 cells were transfected with two different si-RBCK1 oligos or si-Control. After 48 h, total RNA was extracted and RNA-seq analysis was conducted. The global gene expression analysis was based on RNA-seq platform from Beijing Genomic Institute. The RNA-seq data are deposited in the Gene Expression Omnibus (GEO) database (Assessing number: GSE119864). Analysis was performed for differentially expressed genes (*P* < 0.01 and fold change > 2) with Ingenuity Pathway Analysis.

### Co-immunoprecipitation (Co-IP) assay

Cells were harvested in NP-40 lysis buffer containing the protease inhibitor phenylmethane sulfonyl fluoride (PMSF; 1:100). First, 100 μg of cell lysates was pre-cleared with rabbit IgG for 2 h and subsequently incubated overnight with RBCK1 rabbit antibody (ab38540) or rabbit IgG as a negative control. The bound proteins were analyzed with western blotting mouse anti-human p53 antibody (ab1101). For the mutant domain Co-IP experiment, HEK293 cells were transfected with 2 μg of Flag-RBCK1 or its mutants or EGFP-p53 or its mutants plasmids in a 6-cm dish. Cell lysates were pre-cleared with rabbit IgG for 2 h and subsequently incubated overnight with Flag antibody; or rabbit IgG as a negative control. The bound proteins were analyzed with western blotting using relevant antibodies.

### Glutathione S-transferase (GST) pull-down assay

The GST pull-down assay is an effective method to examine the direct binding of two proteins in vitro. This protocol is based on the GST pull-down system from GE Healthcare (4B Glutathione-Sepharose 17075601). The p53 fragment was individually expressed as a His-fusion protein. GST-fused RBCK1 protein was purified using glutathione-Sepharose beads according to the manufacturer’s protocol. Details of synthetic protein sequences are shown in Table S1. The mixture was incubated at 4 °C with rotation for 30 min, and the resin was washed twice with phosphate-buffered saline (PBS) containing 30 mM imidazole, followed by washing twice with PBS containing 0.01% Triton X-100. The bound proteins were eluted with an elution buffer (50 mM sodium phosphate, 300 mM sodium chloride, 250 mM imidazole, pH 7.4) and subjected to SDS-PAGE analysis.

### Protein stability assays

Caki-1 cells were transfected with 100 nM siRBCK1 or siControl. Twenty-four hours post transfection, cells were treated with cycloheximide (CHX, 100 μM) or MG132 (10 μM) for the indicated time points. MG132 is a potent, reversible, and cell-permeable proteasome inhibitor that reduces the degradation of ubiquitin-conjugated proteins in mammalian cells and permeable strains of yeast by the 26S complex. CHX is a eukaryote protein synthesis inhibitor, produced by the bacterium Streptomyces griseus. CHX interferes with the translocation step in protein synthesis and is widely used in biomedical research to inhibit protein synthesis in vitro. Samples were analyzed for p53 degradation by western blotting.

### Immunohistochemistry (IHC) analysis

IHC was used to demonstrate the presence and location of RBCK1 and p53 in renal tissue samples. Formalin-fixed, paraffin-embedded blocks from all patients in the study were obtained from the Department of Renal Cancer and Melanoma, Beijing Cancer Hospital. The blocks were sectioned at a thickness of 4 μm and deparaffinized. First, the sections were incubated in xylene for 20 min, followed by treatment with 100%, 95%, and 75% ethanol. The blocks were washed with water and boiled in ethylenediaminetetraacetic acid (EDTA) buffer for 15 min at 121 °C for antigen retrieval. After being washed with PBS, the sections were incubated in 3% peroxidase for 10 min and washed with water. The sections were blocked with bovine serum albumin (BSA) at room temperature for 20 min, and followed by their exposure to the primary antibodies, anti-RBCK1 (1:200;ab38540, Abcam, Cambridge, MA) or anti-p53 (1:200;ab1101, Abcam, Cambridge, MA) diluted in antibody diluent for 18 h at 4 °C. The sections were incubated with a general secondary antibody at room temperature for 30 min, and then visualized by dextran polymer-conjugated horseradish-peroxidase and 3,3′-diaminobenzidine (DAB) chromogen. The sections were counterstained with hematoxylin solution. Negative control slides in the absence of primary antibodies were included for each staining. The staining intensity and proportion of positive cells were calculated. The intensity was scored as follows, 0 = negative, 1 = weak, 2 = moderate, 3 = strong. Proportion scores are points assigned based on the percentage of positive cells as follows: <10% for 0 point, 10–25% for 1 point. 26–50% for 2 point, 51–75% for 3 point and >75% for 4 point. A total score was obtained by multiplying intensity score × proportion score. The total score ranged from 0 to 12. A total score > 6 was determined as high expression and that less than 6 was considered as low expression. IHC staining was evaluated independently evaluated by different investigators (Sifan Yu and one pathologist).

### Clinical data and tissues

A total of 102 patients with renal cancer that were treated at Peking University Cancer Hospital & Institute between 2008 and 2016 were included in this study (Table 1). The diagnosis of renal clear cell carcinoma was confirmed via identification by the pathology department. Clinical data, including age, sex, AJCC stage, Fuhrman grade and smoking status were collected. This investigation was approved by the Ethics Committee of Peking University and was conducted according to the Declaration of Helsinki Principles. Written informed consent was obtained from each patient prior to the start of research activities.

**Table 1:** Characteristics of 102 patients with renal cancer and expression status of RBCK1 and P53

		P53				RBCK1		
		Cases	High	Low	*P* value	High	Low	*P* value
Gender								
	Male	73	43(58.9%)	30(41.1%)	0.769	39(53.4%)	34(46.6%)	0.157
	Female	29	18(62.0%)	11(38.0%)		11(37.9%)	18(62.1%)	
Age (Years)								
	≤65	69	40(57.9%)	29(42.1%)	0.585	38(55.0%)	31(45.0%)	0.078
	>65	33	21(63.6%)	12(36.4%)		12(36.3%)	21(63.7%)	
Fuhrman								
	I-II	81	50(61.7%)	31(38.3%)	0.436	32(39.5%)	49(60.5%)	<**0.001**
	III-IV	21	11(52.3%)	10(47.7%)		18(85.7%)	3(14.3%)	
AJCC								
	I-II	76	46(60.5%)	30(39.5%)	**0.035**	31(40.8%)	45(59.2%)	**0.004**
	III-IV	26	15(57.7%)	11(42.3%)		19(73.0%)	7(27.0%)	
Smoking status								
	Ever or Current	67	39(58.2%)	28(41.8%)	0.649	36(53.7%)	31(46.3%)	0.487
	Never	35	22(62.8%)	13(37.2%)		14(40.0%)	21(60.0%)	

*Significance evaluated by chi-squared tests

### Glucose measurement

Direct measurement of glucose in cell lysates was performed using Glucose Assay Kit (ab65333, Abcam). The glucose enzyme mix in the kit specifically oxidizes glucose to generate a product, which reacts with a dye to produce a colored compound (570 nm). The intensity of the color is proportional to the amount of glucose present in the sample. Briefly, cells (2 × 10^6^ cells per assay) were harvested and washed with cold PBS. The cells were resuspend in 100 μL of assay buffer and centrifuged for 2 min at 4 °C at maximum speed in a cold microcentrifuge to remove any insoluble material. Supernatant was transferred to a clean tube and left on ice. After being deproteinized, samples or standards at 50 μL concentration were mixed with 50 μL of glucose reaction mix and incubated for 30 min at 37 °C in the absence of light. Absorbance at 570 nm wavelength was meassured on a microplate reader.

### Statistical analysis

SPSS 20.0 software was used for all statistical analyses. Data represent the mean ± standard deviation (SD). We used the Student’s *t*-test and chi-squared test to evaluate differences between two groups. All statistical analyses were two-sided, and a value of *P* < 0.05 was considered statistically significant.

## Results

### RBCK1 is overexpressed in renal cancer and its expression correlates with poor survival in RCC patients

**Fig. 1:**
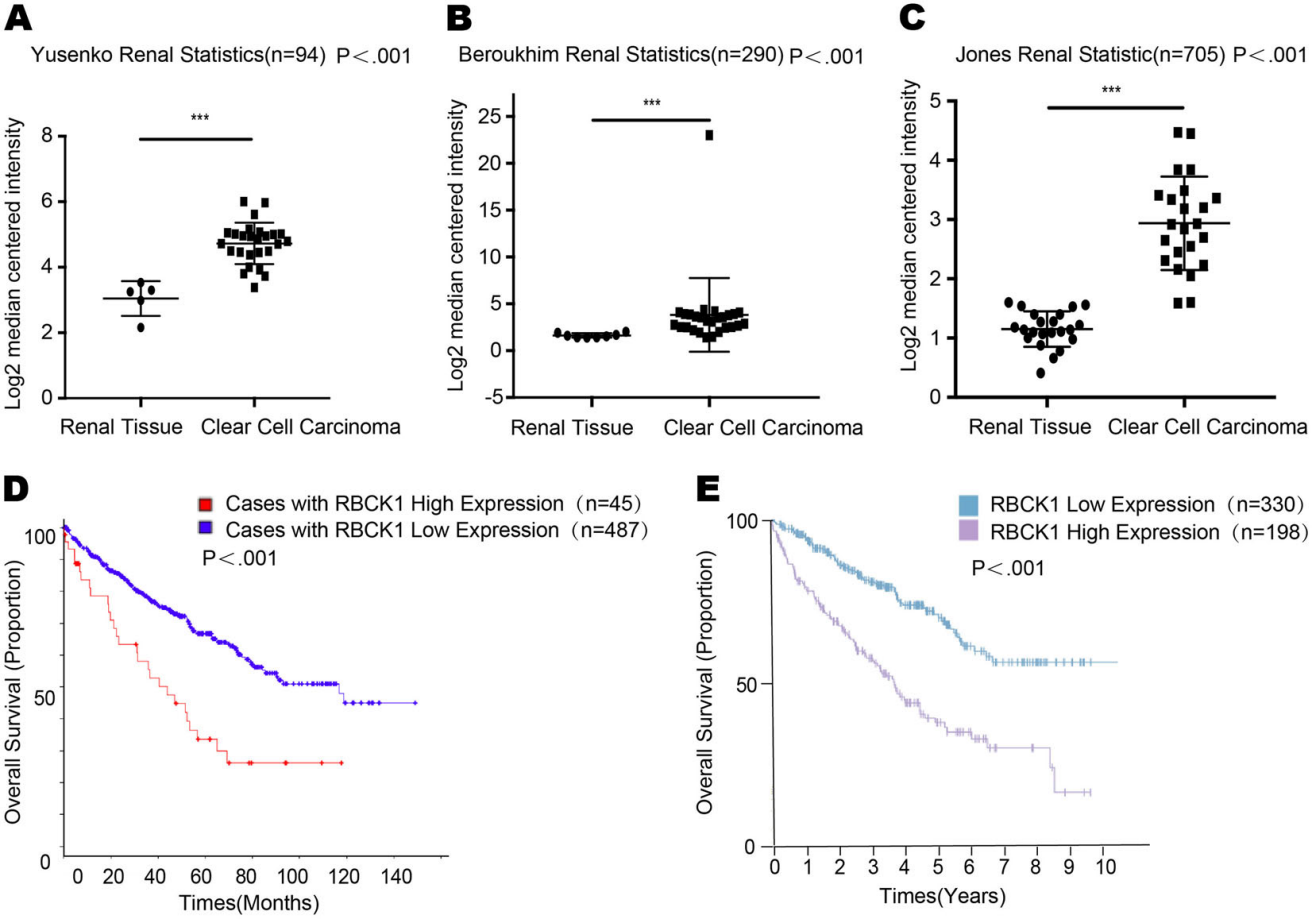
RBCK1 is highly expressed in RCC tissues and correlates with poor prognosis. **a**–**c** RBCK1 mRNA level comparison between renal cancer and no-rmal renal tissue from the Oncomine database. (https://www.oncomine.org/resource/main.html#v:18). *P < .05; **P < .01; ***P < .001. **d** RBCK1 mRNA and expression level correlates with poor prognosis in RCC patients from the TCGA database. (http://www.cbioportal.org/index.do?cancer_study). **e** RBCK1 expression level correlates with poor prognosis in RCC patients from the Proteinatlas database. (https://www.proteinatlas.org/ENSG00000125826)

As shown in Fig. 1, in the three studies of RBCK1 expression available at Oncomine, Yusenko et al.^24^, Beroukhim et al.^25^ and Jones et al.^26^ found that RBCK1 mRNA level in renal cancer was significantly higher compared with normal renal tissue (https://www.oncomine.org/resource/main.html#v:18) (*P* < .001; Figs. 1a–c). A cohort of 532 renal tissues and a cohort of 528 cases were obtained respectively from the The Cancer Genome Atlas (TCGA) database and the Human Protein Atlas databases. Through analysis of the renal cancer survival data, we revealed that patients with high expression of RBCK1 tended to achieve a shorter overall survival time, indicating that RBCK1 expression correlated with poor prognosis in RCC patients. (http://www.cbioportal.org/index.do?cancer_study) (https://www.proteinatlas.org/ENSG00000125826) (P < .001; Fig. 1d, e)

### RBCK1 depletion affects renal cancer cell proliferation, apoptosis, and cell cycle arrest

**Fig. 2:**
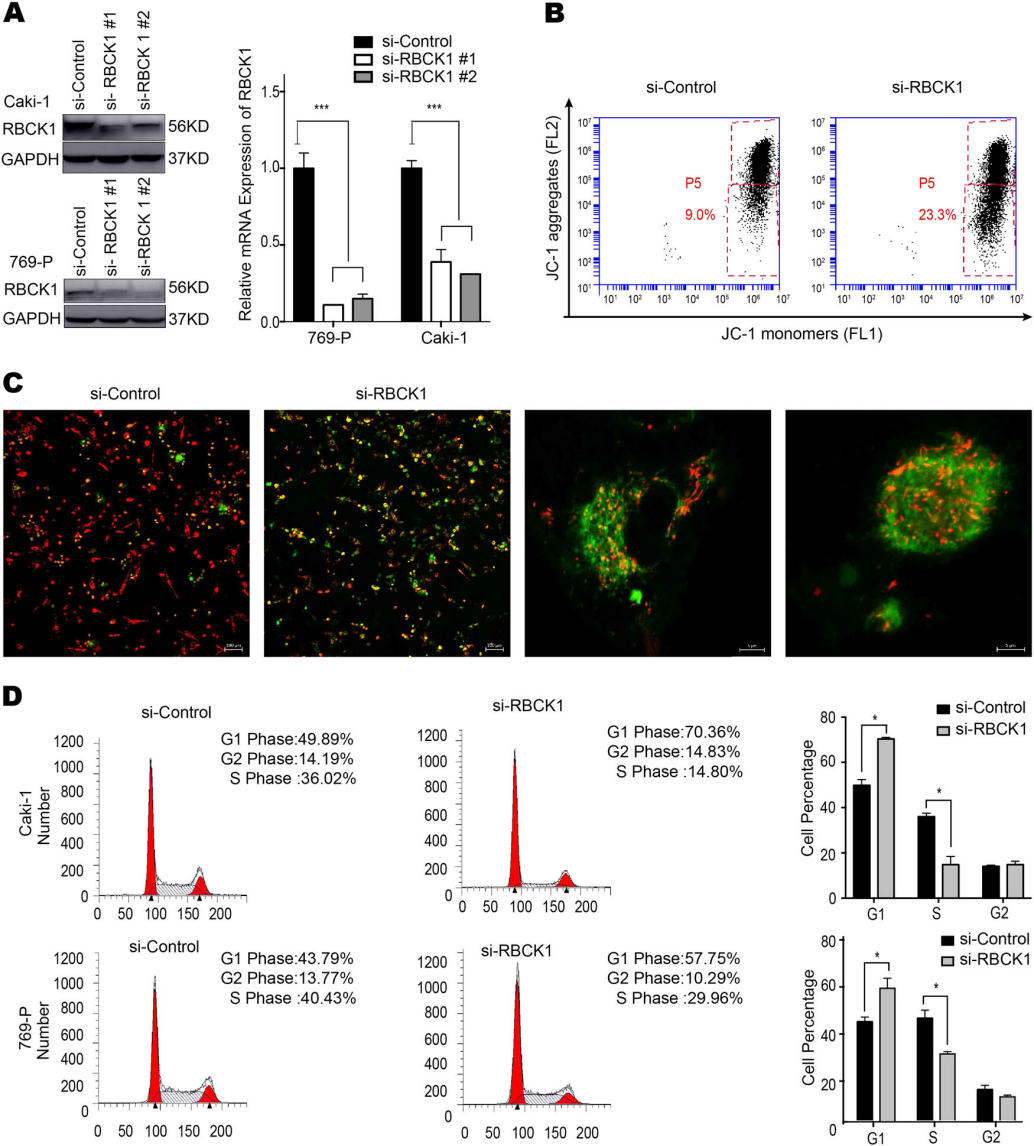
RBCK1 depletion affects RCC apoptosis and cell cycle aggression. **a** RBCK1 knockdown efficiency by two different siRBCK1 oligos in Caki-1 cells and 769-P cells. siControl was compared to siRBCK1 #1 group/siRBCK1 #2 group separately. ***P < .001. **b** Flow cytometry was used to evaluate the mitochondrial membrane potential (MMP). Data were revealed as the monomers positive percentage of the treated cells to that of the untreated control. ***P < .001. **c** Cells containing J-aggregates have high MMP, and show red fluorescence (590 nm, FL-2 channel). Cells with low MMP are those in which JC-1 maintains monomeric form, and show green fluorescence (530 nm, FL-1 channel). Caki-1 cells were transfected with 50 nM siRBCK1 or control. After 24 h, JC-1 exhibits potential-dependent accumulation in mitochondria, indicated by a fluorescence emission shift from green to red. **d** Cell cycle distribution was evaluated by flow cytometry. Caki-1/769-P cells were transfected with RBCK1 siRNA or control siRNA (*P* < 0.05). Experiments were done in triplicate

**Fig. 3:**
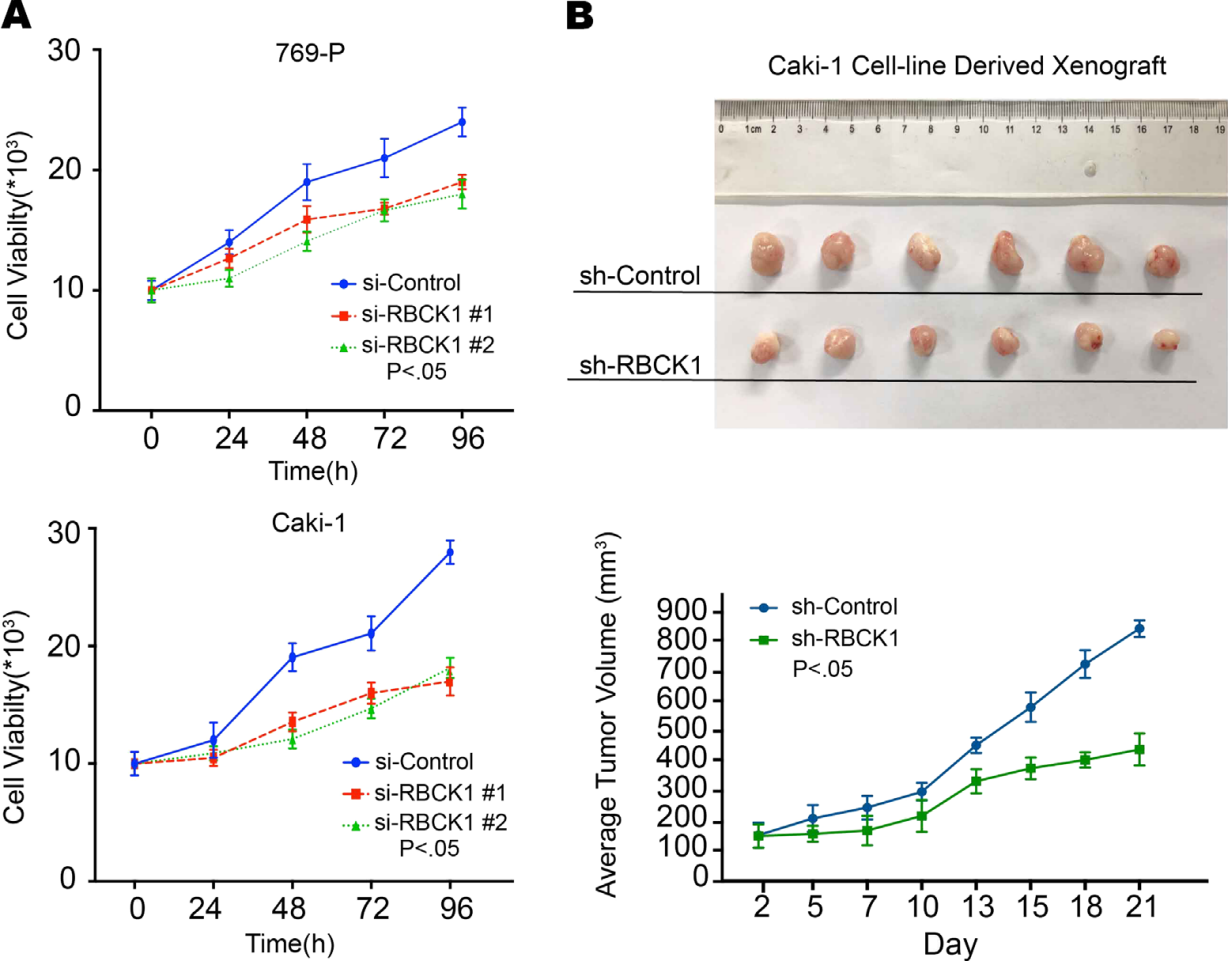
RBCK1 depletion inhibits RCC proliferation in vivo and in vitro. **a** The CCK8 assay was used to determine the cellular metabolic activity at indicated time points after transfection. Experiments were done in triplicates in two kinds of cell-lines of renal cancer. All values are mean ± SD (*n* = 3, *P < .05). **b** Caki-1 cells were stably transfected with lentivirus carrying scramble shRNA or RBCK1 shRNA. NOD-SCID mice were used. The mice were subcutaneously inoculated with cells (5 × 10^6^ Caki-1 cells suspended in 100 ul PBS solution) in the logarithmic growth period. At 30 days post-injection, tumor growth was monitored every 2~3 days and the tumor volume were calculated by length × width^2^/2. The mice were sacrificed at 50 days after transplant. The tumor growth curve photograph is shown. (*P* < .05)

To clarify the biological function of RBCK1 in RCC, we transfected the RBCK1-specific siRNA into RCC cell lines Caki-1 and 769-P. RBCK1 inhibition was confirmed at the protein and mRNA levels (Fig. 2a). Breakdown of mitochondrial membrane potential (MMP) is often associated with early apoptosis. Alterations in MMP were analyzed by flow cytometry using JC-1 dye. The percentage of early apoptotic cells was significantly higher in RBCK1 knockdown group than that in the control group (*P* < .05; Fig. 2b). As shown in Fig. 2c, siControl group demonstrated undamaged nucleated cells with high MMP and red fluorescence. On the contrary, RBCK1 depletion resulted in a decrease in MMP; as a result, JC-1 maintained its monomeric form and showed more green fluorescence. We conducted flow cytometric analysis to evaluate whether RBCK1 expression affects cell cycle progression. As shown in Fig. 2d, RBCK1 expression knockdown led to an increased in the proportion of cells in G0/G1 phase (*P* < .05) and decreased the proportion of cells in G2/M. Cell proliferation was significantly decreased following RBCK1 silencing as compared with the control cells (*P* < .05; Fig. 3a). We also assessed whether RBCK1 affects tumor formation in vivo. We successfully constructed stable RBCK1-silencing clones of Caki-1 cells and carried out tumorigenicity assays in 12 NOD-SCID mice with subcutaneously injected with 5 × 10^6^ Caki-1-shRBCK1 or control cells. Consistent with the in vitro results, RBCK1 depletion significantly decreased the rate of tumor growth as compared with the control group (*P* < .05; Fig. 3b). Glucose is a very important fuel source to generate universal energy molecules. To test the effects of RBCK1 depletion influence on metabolism disorder, we conducted glucose assay using two RCC cell lines. The intracellular glucose level was analyzed after cell lysis. We found that the depletion of RBCK1 expression caused an increase in the intracellular level of glucose, suggesting that RBCK1 may affect the efficiency of glucose utilization and decrease the glucose consumption that may eventually lead to metabolic disorders in RCC cells (*P* < .05; Supplementary Fig. 1A, B).

### RBCK1 depletion increases the expression of p53 target genes in renal cancer cells

**Fig. 4:**
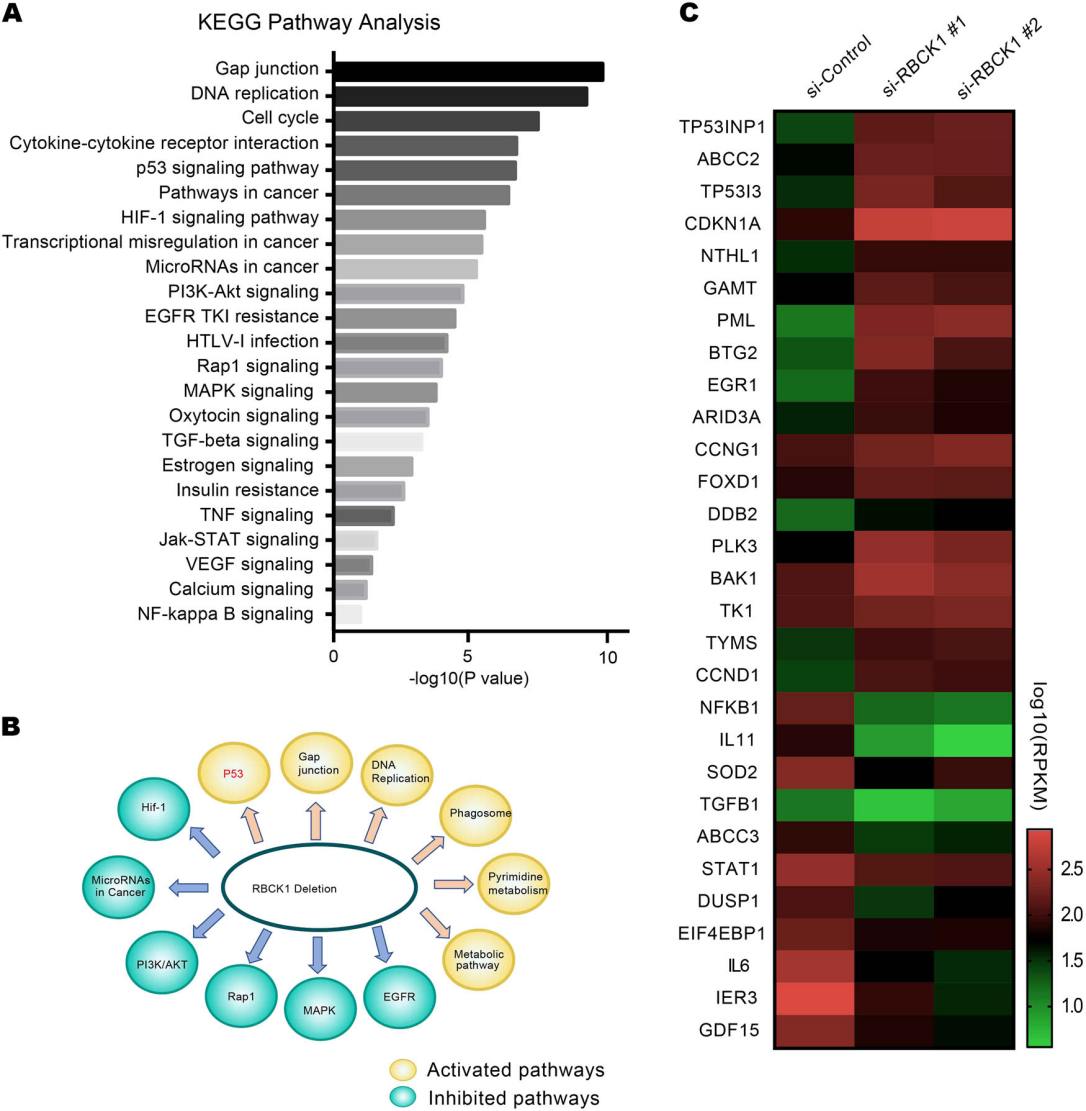
RBCK1 regulates p53 signaling. **a** The 23 most significantly enriched KEGG pathways analysis. The bars represent significance as −log (*p*-value). All ontology enrichments were filtered to allow no more than a 10% false discovery rate. **b** Schematic graph illustrates significantly changed signaling by RBCK1 depletion in Caki-1 cells. Signal pathway enrichment analysis was used to derive the related pathways, using *P* < .01 and fold change > 2 as a cutoff to derive regulated genes, and *P* < .001 to define significantly enriched pathways. **c** The heatmap graph shows the p53-activated genes in Caki-1 cells by RBCK1 knockdown

To approach the function of RBCK1 in RCC cells in an unbiased way, we analyzed the changes in RNA-seq data following RBCK1 depletion in Caki-1 cells with the GEO accession number GSE119864. Analysis of the 23 most significantly enriched Kyoto Encyclopedia of Genes and Genomes (KEGG) pathways was performed (Fig. 4a). The pathway analysis revealed that RBCK1 depletion downregulated the activity of several pathways, including mitogen-activated protein kinase, epidermal growth factor receptor and phosphoinositide 3-kinase, and activated some other pathways, such as the p53 pathway (Fig. 4b). Considering the important role of p53 as a tumor suppressor in cancers, we set out to study the expression of p53 downstream target genes. As a result, we found that the expression of a group of target genes activated by p53 was upregulated, including *CDKN1A* (also known as P21) and *TP53INP1*, while the genes that are suppressed by the p53 were downregulated, such as *ABCC3* and *SOD2* (Fig. 4c).

**Fig. 5:**
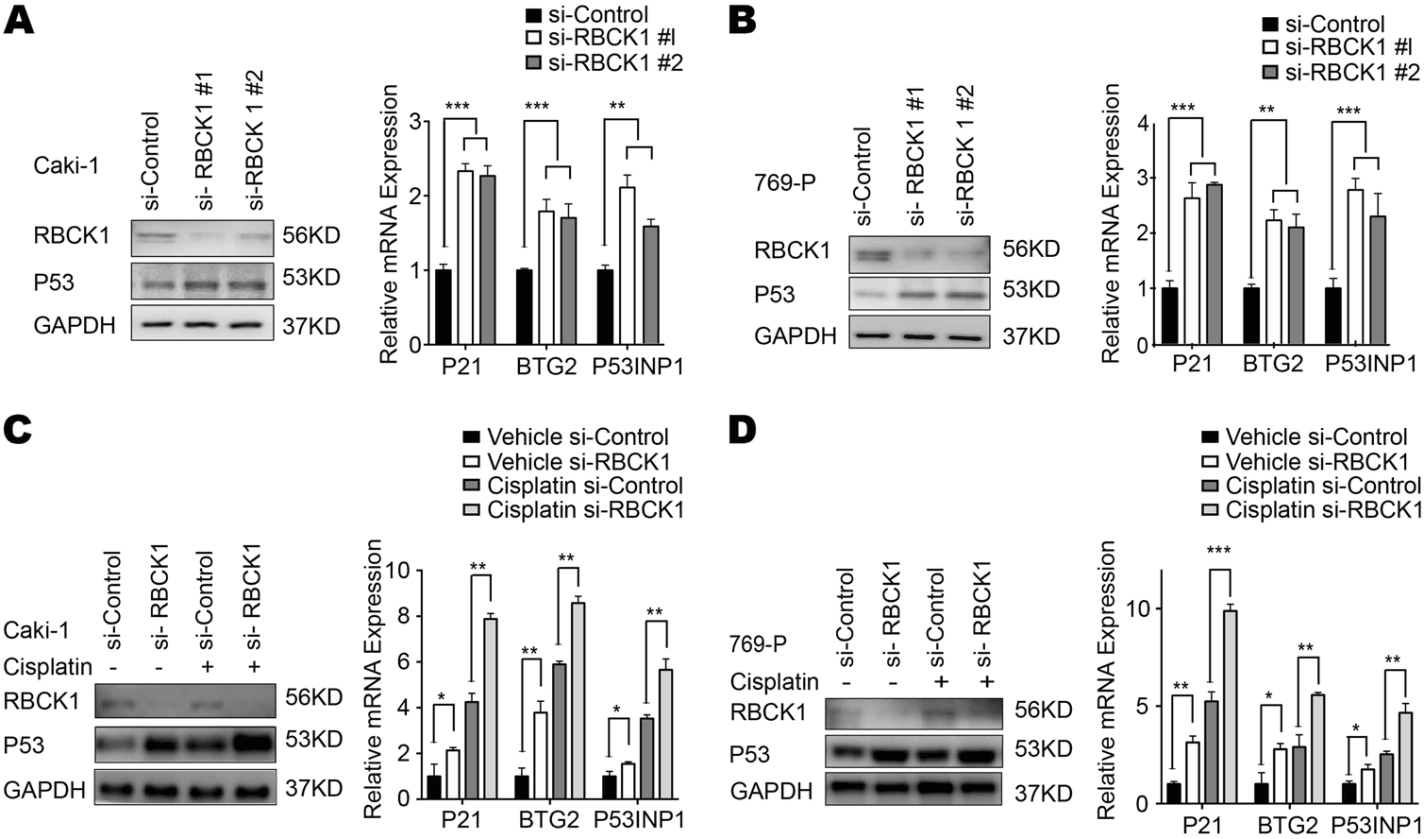
RBCK1 controls p53 protein levels and expression of p53 target genes. **a** RBCK1 depletion increases p53 protein levels and p53 target genes using two different siRNA oligos. siControl was compared to siRBCK1 #1 group/siRBCK1 #2 group separately. After 48 h, p53 and RBCK1 levels were determined by Western blot analysis and RNA was prepared for detecting endogenous p53 target genes, P21, P53INP1 and BTG2 by qPCR. Shown are the results from three experiments. *P* value for siRBCK1 versus siControl. GAPDH was used as the internal control. **P < .01; ***P < .001. **b** RBCK1 depletion increases p53 protein levels and p53 target genes using two different siRNA oligos. siControl was compared to siRBCK1 #1 group/siRBCK1 #2 group separately. After 48 h, p53 and RBCK1 levels were determined by Western blot analysis and RNA was prepared for detecting endogenous p53 target genes, P21, P53INP1 and BTG2 by qPCR. Shown are the results from three experiments. *P* value for siRBCK1 versus siControl. GAPDH was used as the internal control. **P < .01; ***P < .001. **c** RBCK1 depletion increases p53 protein levels and p53 target genes under the treatment of cisplatin. After 48 h of transfection, cells were treated with 10 μM cisplatin or vehicle. p53 and RBCK1 levels were determined by Western blot analysis. Each experiment was repeated three times and GAPDH was used as the internal control. The expression levels of the endogenous p53 target genes, P21, P53INP1 and BTG2 were determined by qPCR. 48 h after transfection, cells were treated with 10 μM cisplatin or vehicle for 6 h and RNA was prepared. Shown are the results from the triplicate experiments. *P* value for siRBCK1 versus siControl. siControl were compared to sRBCK1 group; in cisplatin treated samples, siControl was compared to the siRBCK1 group separately. *P < .05; **P < .01; ***P < .001. **d** RBCK1 depletion increases p53 protein levels and p53 target genes under the treatment of cisplatin. 48 h after transfection, cells were treated with 10 μM cisplatin or vehicle. p53 and RBCK1 levels were determined by Western blot analysis. Each experiment was repeated three times and GAPDH was used as the internal control. The expression levels of the endogenous p53 target genes, P21, P53INP1 and BTG2 were determined by qPCR. 48 h after transfection, cells were treated with 10 μM cisplatin or vehicle for 6 h and RNA was prepared. Shown are the results from the triplicate experiments. *P* value for siRBCK1 versus siControl. siControl were compared to sRBCK1 group; in cisplatin treated samples, siControl was compared to the siRBCK1 group separately. *P < .05; **P < .01; ***P < .001

To confirm this RNA-seq and in silico findings, we further investigated the expression of p53 and its targets in the two cell lines expressing wild-type p53. As shown in Figs. 5a, b, RBCK1 depletion increased the expression of p53 protein level and its target genes, including *P21*, *BTG2* and *P53INP1*. To confirm the suppressive effect of RBCK1 on p53 signaling, we treated the two RCC cell lines with cisplatin. Cisplatin causes DNA damage and subsequently activates p53 signaling. The results of western blot analysis shown in Figs. 5c, d reveal the increase in the expression of p53 in both cell lines following depletion. Following treatment with cisplatin, p53 protein level further enhanced. In addition, an increase in the expression of p53 target genes *P21*, *BTG2* and *P53INP1* was also observed after the depletion of RBCK1. These effects further enhanced after RBCK1 depletion in cisplatin-induced condition (Fig. 5c, d).

**Fig. 6:**
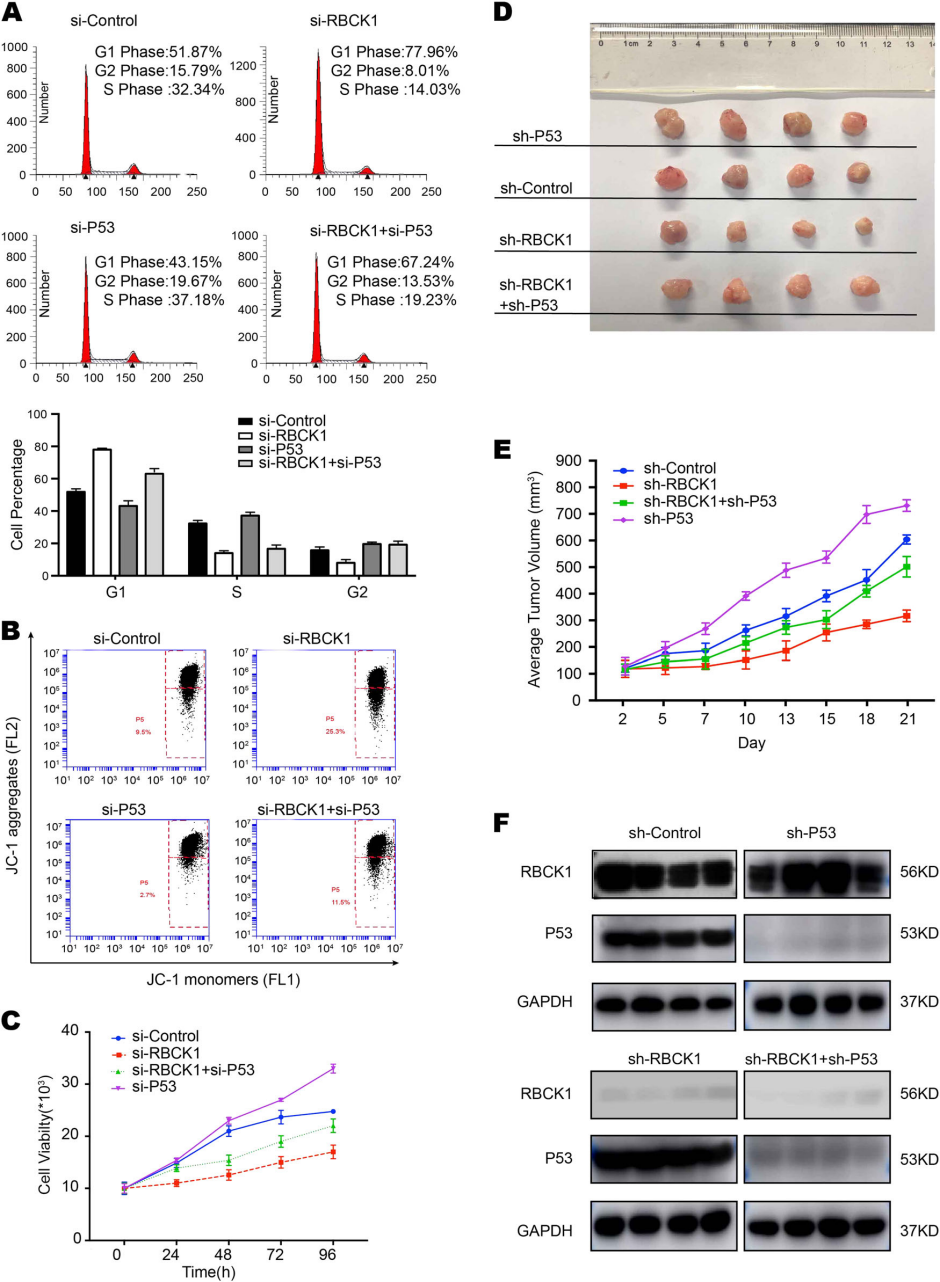
Biological behavior affected by RBCK1 depletion could be rescued by p53 silencing in vivo/vitro. **a** Cell cycle distribution was evaluated by flow cytometry. Caki-1 cells were transfected with control, RBCK1 siRNA,p53 siRNA or both types of siRNA (*P* < 0.05). Experiments were done in triplicate. **b** Flow cytometry was used to evaluate the MMP in four groups. Data were revealed as the monomers positive percentage of the treated Caki-1 cells to that of the untreated control. *P* < .05. **c** The CCK8 assay was used to determine the cellular metabolic activity at indicated time points after transfection. Experiments were done in triplicates in Caki-1 cells which were divided into four groups. All values are mean ± SD (*n* = 3, *P < .05). **d** Caki-1 cells were stably transfected with lentivirus carrying scramble sh-p53 or sh-RBCK1 or both shRNA. 16 NOD-SCID mice were divided into four groups. The mice were subcutaneously inoculated with cells (5 × 10^6^ cells suspended in 100 ul PBS solution) in the logarithmic growth period. At 28 days post-injection, tumor growth was monitored every 2~3 days and the tumor volume were calculated by length × width^2^/2. The mice were sacrificed at 50 days after transplant. **e** The tumor growth curve photograph is shown. (*P* < .05). **f** Western blotting was conducted to determine the persistence of RBCK1/p53 silencing in tumor masses in 16 mice tumor tissues. GAPDH was used as an internal control

To increase the credibility of the results, we simultaneously silenced p53 and RBCK1 expression in RCC cell lines. As shown in Fig. 6a, RBCK1 depletion alone led to an increased in the proportion of cells in the G0/G1 phase and decreased the proportion of cells in G2/M phase. G1 cell cycle arrest was significantly improved after the silencing of RBCK1 and p53 expression. The percentage of early apoptotic cells was significantly higher in double silencing group than in cells lacking RBCK1 expression only (Fig. 6b). In comparison with the cells treated with si-RBCK1, those treated with si-RBCK1 and si-p53 showed an increase in the proliferation ability in vivo and in vitro (Fig. 6c–e). These results shows that the altered biological behaviors caused by RBCK1 depletion could be partially reversed via p53 gene silencing, indicative of the effects of RBCK1 on RCC cells via p53.

### Intracellular localization of RBCK1 and its interaction with p53

**Fig. 7:**
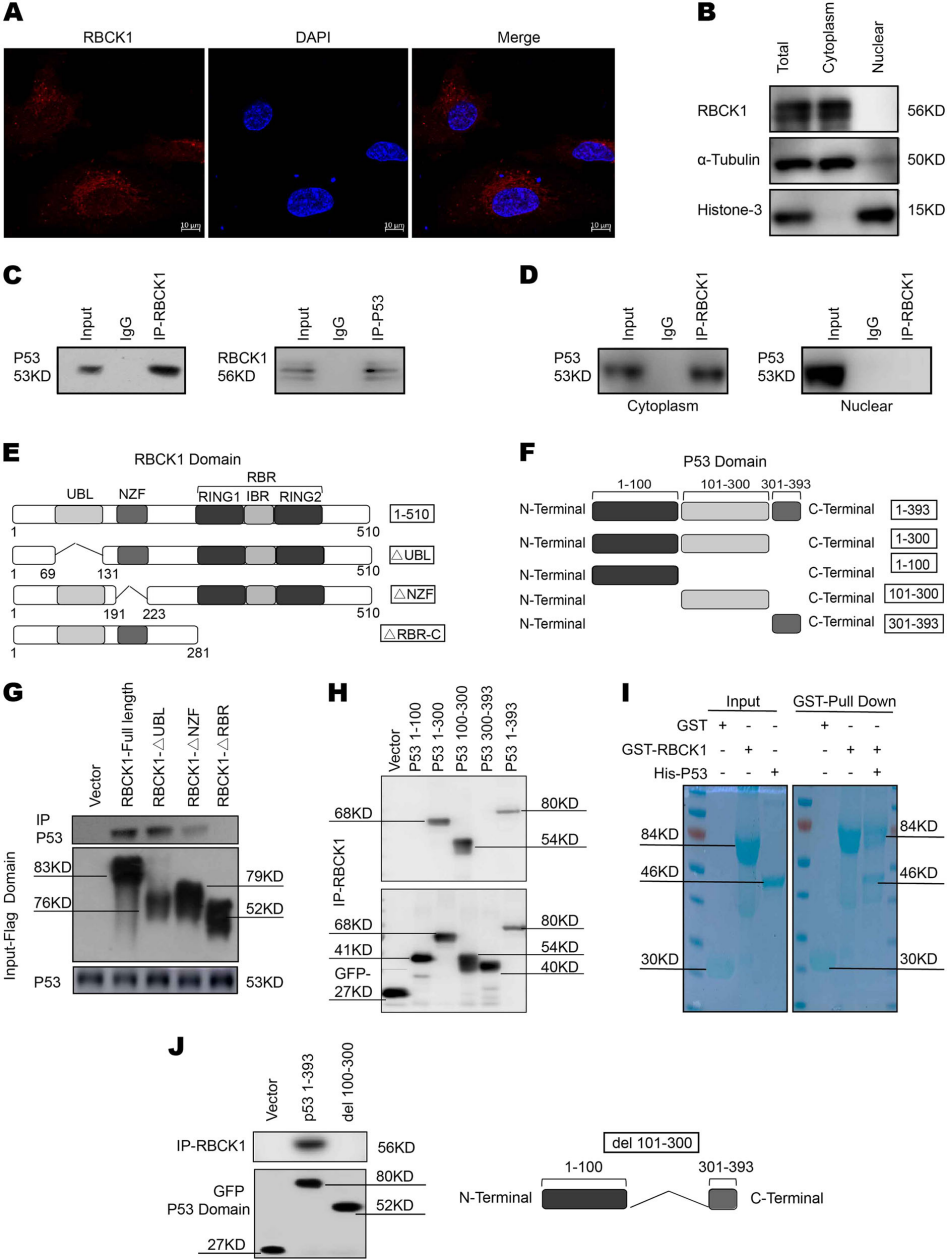
Intracellular localization of RBCK1 and its interaction with p53. **a** Intracellular localization analysis of RBCK1 by immunofluorescence assay. Caki-1 cells were cultured in phenol red-free DMEM medium. Intracellular localization of RBCK1 (red) were shown. Nuclei (blue) were stained with 4′,6-diamidino-2-phenylindole (DAPI). **b** RBCK1 is mainly localized in the cytoplasm. The subcellular protein fractionation kit (Thermo scientific, 78840) was used for cytoplasm and nuclear separation. Tubulin and Histone-3 were used for cytoplasm and nuclear control. **c** Co-IP assays reveal associations between endogenous RBCK1 and p53 in Caki-1 cells. IgG was used as a control. Caki-1 cells were harvested with NP-40 lysis buffer. CO-IP was performed using antibody as indicated. **d** Nuclear and cytoplasmic separation-based Co-IP. The subcellular protein fractionation kit was used for cytoplasm and nuclear separation. Based on the separation, IP was done by RBCK1 antibody in both the cytosol and nuclear lysis. p53 antibody was used to detect the interaction in both the cytosol and nuclear. **e** RBCK1 domain structure and deletion mutants used in the study (Full length, ΔUBL, ΔNZF, ΔRBR-C). **f** P53 domain structure and deletion mutants used in the study (Full length, aa1–300, aa1–100, aa101–300, aa301–393). **g** HEK293 cells were transfected with 2 μg EGFP-p53 together with Flag-RBCK1 full length or mutants (ΔUBL, ΔNZF, ΔRBR-C). After 24 h, cells were harvested with NP-40 lysis buffer. Co-IP was performed using EGFP antibody. The possible interacted RBCK1 domains were detected by flag antibody. **h** HEK293 cells were transfected with 2 μg Flag-RBCK1 together with EGFP-p53 full length or mutants (aa1–300, aa1–100, aa101–300, aa301–393). After 24 h, cells were harvested with NP-40 lysis buffer. Co-IP was performed using flag antibody. The possible interacted p53 domains were detected by EGFP antibody. **i** Pulldown assay experiment. p53 fragment was individually expressed as His-fusion protein. GST-fusion RBCK1 protein was purified using glutathione-Sepharose beads according to the Manufacturer’s protocol. **j** Co-IP shows that mutants p53 del 100–300 fails to interact with RBCK1. HEK293 cells were transfected with 2 μg Flag-RBCK1 together with EGFP-p53 full length or del 100–300 mutants. After 24 h, cells were harvested with NP-40 lysis buffer. Co-IP was performed using flag antibody

Immunostaining results showed that RBCK1 was mainly localized in the cytosol (Fig. 7a), consistent with the results of western blot analysis with separated nuclear and cytoplasmic proteins (Fig. 7b). The functional cooperation between RBCK1 and p53 was further confirmed by the results of Co-IP using the endogenous proteins from Caki-1 cells. Co-IP analysis showed that RBCK1 could precipitate together with p53 (Fig. 7c). Nuclear and cytoplasmic separation-based Co-IP demonstrated that RBCK1 interacted with p53 in the cytoplasm (Fig. 7d). RBCK1 exhibits three functional domains, namely, the UBL domain, NZF domain and a catalytic C-terminal RBR (RING-IBR-RING) domain. p53 has several functional domains, including a transactivation and proline rich domain (aa1–100), DNA-binding domain (aa101–300) and tetramerization and C-terminal domain (aa301–393) (Fig. 7e, f). We developed deletion constructs in order to delineate the interaction between RBCK1 and p53. Full-length of RBCK1 or its deletion constructs (ΔUBL domain, ΔNZF domain and ΔRBR-C domain) were expressed together with p53 in HEK293 cells. Co-IP assay results indicated that the RBR-C domain was essential for the interaction of RBCK1 with p53 (Fig. 7g). In addition, the full-length of p53 or deletion constructs (aa1–300, aa1–100, aa101–300, aa301–393) were expressed together with RBCK1 in HEK293 cells. Co-IP assay showed that the DNA-binding domain (aa101–300) of p53 was necessary for its interaction with RBCK1 (Fig. 7h). *Escherichia coli*-based protein expression coupled with pull-down assay also detected the direct interaction between RBCK1 and p53 (Fig. 7i). To further confirm the domain responsible for the interaction between p53 and RBCK1, we constructed a plasmid missing DNA-binding domain and failed to observe any interaction between p53 and RBCK1 in the Co-IP assay (Fig. 7j).

### RBCK1 modulates p53 protein stability

**Fig. 8:**
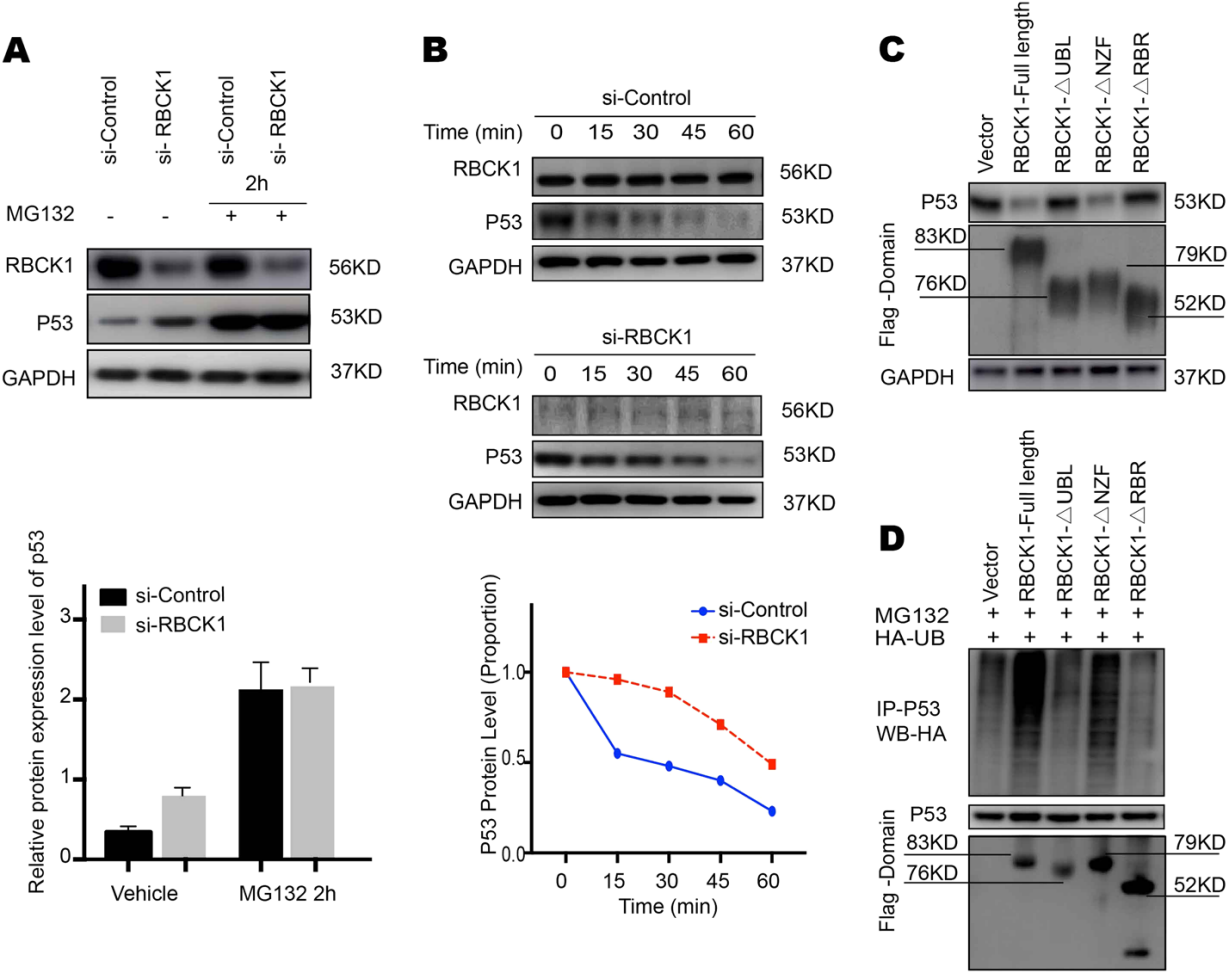
RBCK1 associates with p53 and decreases p53 stability. **a** RBCK1 promotes proteasome-mediated p53 degradation. After 24 h of transfection, cells were treated with 10 μM MG132/vehicle for the indicated time. Cell lysis was prepared for Western blot analysis. The following Image is a histogram of the relative expression of p53 protein. **b** RBCK1 decreases p53 half-life in RCC cells. Caki-1 cells were transfected with siControl and siRBCK1. After 24 h, cells were treated with 100 μM cycloheximide/vehicle for indicated times. Cell lysates were prepared for Western blot analysis. Image J was used to quantify the p53 protein level. For p53 density, each cycloheximide treated time point was normalized to its zero time point for each group. **c** RBCK1 UBL and/or RBR domain decreases p53 stability. HEK293 cells were transfected with vector or different RBCK1 deletion mutant plasmids. After 24 h, cell lysis was prepared for Western blot analysis. **d** HEK293 cells were transfected with 2 μg EGFP-p53 plasmid, 0.5 μg HA-Ub plasmid and 0.5 μg flag-RBCK1/ flag -domain/flag -vector plasmids. The cell extracts were immunoprecipitated with p53 antibody. The poly-ubiquitinated p53 was detected by HA antibody

To explore the potential mechanism underlying RBCK1-mediated regulation of p53 expression, we performed a series of protein stability assays. MG132 is a peptide aldehyde that effectively blocks the proteolytic activity of the 26 S proteasome complex. Caki-1 cells were transfected with si-Control or si-RBCK1. After 24 h, the cells were treated with 10 μM of MG132 or vehicle for the indicated time points. Cells treated with MG132 showed an increased in the expression of p53 within 2 h. The upregulation in the expression of p53 caused by RBCK1 depletion was almost eliminated (Fig. 8a). CHX is widely used to inhibit protein synthesis in eukaryotic cells. Following treatment with CHX, the RBCK1-depleted cells showed a significant increase in the half-life of endogenous p53 (*P* < .05; Fig. 8b). We overexpressed the full-length or deletion constructs (ΔUBL domain, ΔNZF domain and ΔRBR-C domain) of RBCK1 in HEK293 cells and found that the UBL and/or RBR domain were essential for mediating RBCK1 stabilization effect on p53 (Fig. 8c). Co-IP analysis of cell lysate from HEK293 cells co-transfected with p53, HA-Ub, and RBCK1 or its different domain constructs revealed that RBCK UBL and/or RBR domain are necessary for mediating the inhibitory effects of on p53 poly-ubiquitination. As a ubiquitin ligase, RBCK1 possibly exerts its function in a ubiquitin-based manner (Fig. 8d).

### High RBCK1 expression and low p53 expression predict high staging risk for renal cancer patients

**Fig. 9:**
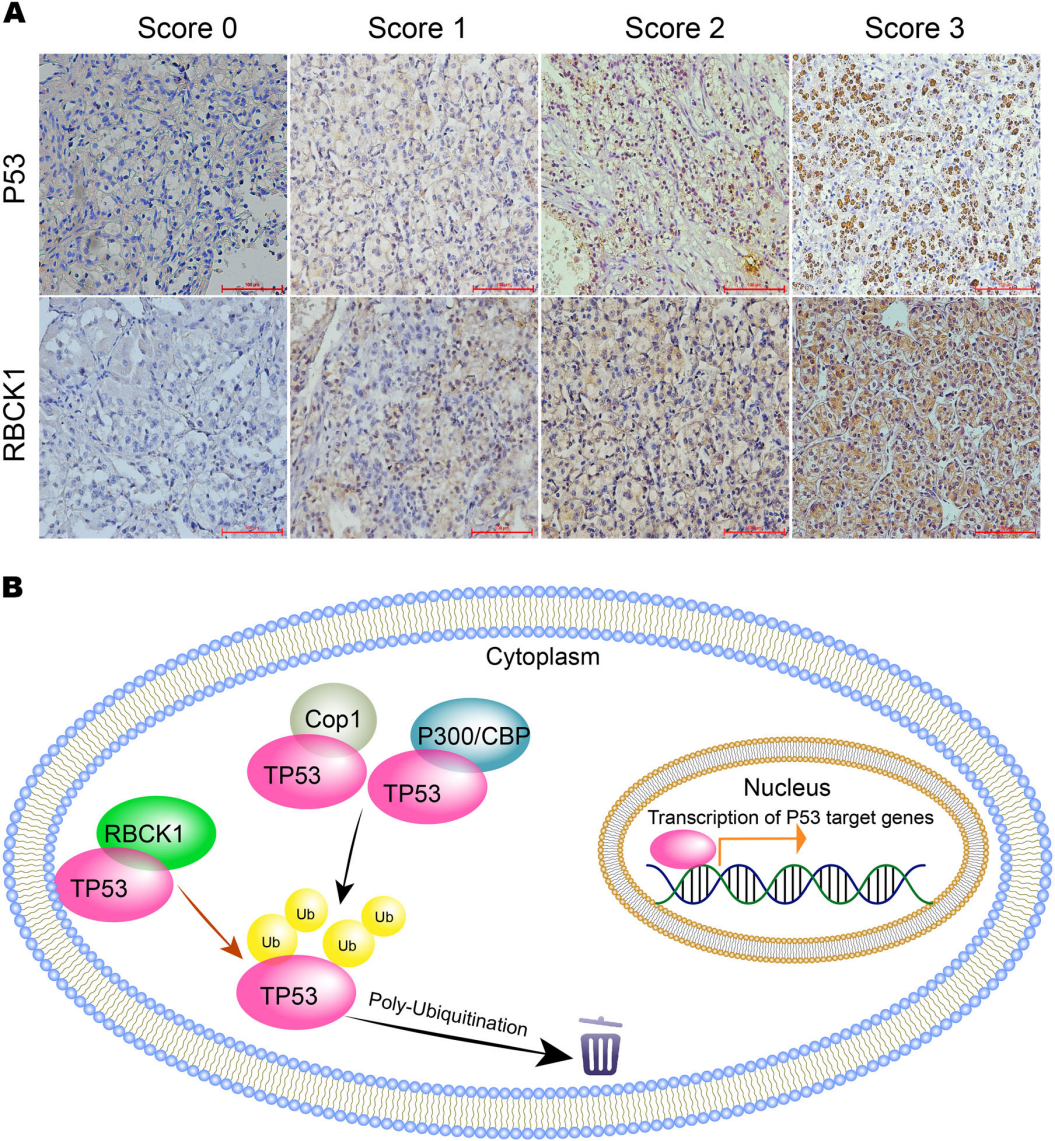
Immunohistochemistry examination of RBCK1 and p53 expression in RCC tissues. **a**, **b** The hypothetical model for the functional interplay of RBCK1 with p53 in renal cancer cells

We performed IHC staining of tumor tissues to detect RBCK1 and p53 expression in ccRCC patients. The staining of RBCK1 was mainly located in the cytoplasm of the tumor cells, while p53 expression was detected both in the membrane and cytoplasm. A typical staining is shown in Fig. 9a. Based on the RBCK1 staining scores of the tumors, 50 of the enrolled 102 patients were categorized under high expression group, while the other 52 were grouped under low expression. Patients and their clinical characteristics are summarized in Table 1. No statistically significant differences were observed between the groups with respect to age, gender or smoking status. Patients with high RBCK1 expression tended to have advanced TNM stage renal cancer (*P* = .004) and higher Fuhrman grade (*P* < .001), indicating that RBCK1 may be useful in predicting the staging of renal cancer. Patients with low p53 expression showed correlation with advanced stages (*P* = .035).

## Discussion

In the present study, we suggested the relationship between ubiquitin protein RBCK1 and p53. Based on the results established herein, we proposed a model for to promote ubiquitination and degradation of p53 (Fig. 9b). The depletion of RBCK1 in RCC cancer cells may induce p53 expressions and arrest cell cycle progression, suggestive of its potential as a therapeutic target for RCC therapy.

RBCK1 belongs to the family of RBR (RING-IBR (In between RING)-RING) E3 ubiquitin ligases. The RBR motif is typical of an important group of 13 human RBR E3 ubiquitin ligases^27,28^. RBRs use a hybrid mechanism that combines the functions of both RING and HECT E3 ligases to facilitate the ubiquitination reaction. We found that RBCK1 could not only interact with p53, but it can also regulate the protein stability of p53 by ubiquitination. This confirms our hypothesis that the phenotypic changes induced by RBCK1 depletion, cell proliferation and cell cycle arrest are most likely attributable to the degradation of p53 protein. In terms of cell cycle, we observed that most RCC cells were arrested at G1 phase and the p21 protein expression significantly increased after RBCK1 depletion. Several studies have confirmed that p21 as an important target genes of p53 that may induce G1 cell cycle arrest^29^. Our results are consistent with the previously established reports.

RBCK1 acts in tandem with other two ubiquitin ligases RNF31 and SHARPIN to form a 600 kDa linear ubiquitin chain assembly complex (LUBAC), which generates multiple and different ubiquitin chains to exercise the essential functionality of linear ubiquitination^30^. The LUBAC-catalyzed linear ubiquitination of the nuclear factor κB essential modulator (NEMO), a key regulator of the IκB kinase (IKK) complex, results in the induction of oligomerization of the IKK complex^31,32^. Aside from NEMO, several proteins such as RIP1, RIP2 and MyD88, which are involved in nuclear factor kappa B (NF-κB) activation are known as the substrates for LUBAC^33,34^. LUBAC is involved in NF-κB signaling and regulation of cell death^35,36^. Mice lacking LUBAC ligase activity exhibited high cell death rate, suggestive of embryonic lethality^37,38^. In breast cancer, RBCK1 expression was shown to positively correlates with estrogen receptor alpha and the downstream expression of target genes as well as the proliferation of breast cancer cells via interaction with the estrogen receptor alpha promoter^39^. Considering the positive correlation between RBCK1 expression and estrogen receptor alpha, RBCK1 was proposed RBCK1 as a modulator of estrogen receptor alpha expression and a therapeutic target candidate. The other two components of the LUBAC complex have also been shown to regulate the stability of p53 protein. It was proposed that SHARPIN interacts with and stabilizes MDM2, which subsequently promotes p53 poly-ubiquitination and degradation^40^. In line with this model, another research group demonstrated the RNF31-mediated suppression of p53 target genes via stabilization of MDM2^41^.

The protein p53 could be activated by several events such as DNA damage, oxidative stress and oncogene activation^42^. After activation, the half-life of p53 increases leading to the upregulation of activation of p53 target genes^43^. p53 induces protein expression by inhibiting cell cycle progression or promoting cell apoptosis^44,45^. Several p53 target genes, including p21, are involved in cell cycle arrest. Another group of target genes, including those encoding BAX and Fas proteins, regulate cell apoptosis^46^. p53 is also reported to mediate DNA repair via interaction with DNA repair proteins, such as breast cancer gene 1 (BRCA1) and ataxia-telangiesctasia mutated (ATM)^47,48^ and undergoes several types of protein modifications, including phosphorylation, ubiquitination, acetylation and methylation. These processes are tightly related to its physiological function^49^. It is well accepted that the ubiquitin-proteasome pathway plays a major part in regulation of p53 functions. For instance, protein mouse double-minute 2 protein (MDM2) is a negative regulator of p53. After binding to p53, MDM2 inhibits its transcriptional activity, favors its nuclear export, and eventually stimulates its degradation^50^. Researchers have discovered new ubiquitin ligases that could regulate p53 expression^51–54^. Considering p53 inactivation as a key step in the development of several cancer types, restoring p53 function has been the key objective in the field of cancer drug development. To mimic the residues of Phe19, Trp23 and Leu26 residues in p53 and their interaction with MDM2, a series of small molecule MDM2 antagonists have been developed^55^. Different strategies have been adopted in this direction, including the use of the MDM2 antagonists; however, no relevant drug has been able to to achieve satisfactory effects in renal cancer.

The development of drugs targeting p53 and the treatment of RCC have not reached satisfactory outcomes, probably owing to the lack of the proper understanding of the regulation of p53 in RCC. In this direction, we have found a new E3 ligase, RBCK1, which may likely reverse this situation. RBCK1 could reduce the protein stability of p53 protein, and restore its function by targeting RBCK1, thereby serve as a unique and selective target for the discovery of new drugs against renal cancer.

## Supplementary information

Supplement Figure 1

Table S1

Supplemental Figure legends
