# Supplementary figures and images for: RBCK1 promotes p53 degradation via ubiquitination in renal cell carcinoma
Source: Cell Death Dis. 2019 Mar 15;10(4):254. doi: 10.1038/s41419-019-1488-2 (PMC6420644; doi:10.1038/s41419-019-1488-2)

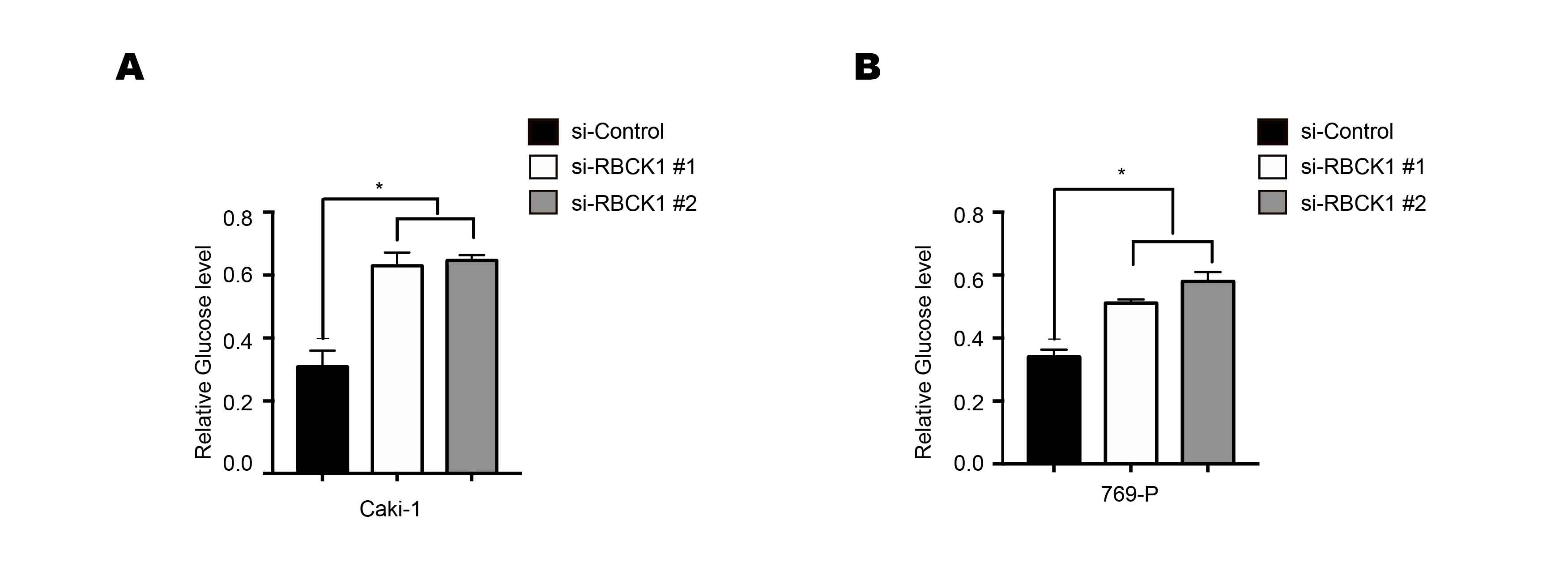

Supplement: Supplementary file 1 — Supplement Figure 1 [file 41419_2019_1488_MOESM1_ESM.jpg]
