# Supplementary material for: RBCK1 promotes p53 degradation via ubiquitination in renal cell carcinoma
Source: Cell Death Dis. 2019 Mar 15;10(4):254. doi: 10.1038/s41419-019-1488-2 (PMC6420644; doi:10.1038/s41419-019-1488-2)
Supplement: Supplementary file 2 — Table S1 [file 41419_2019_1488_MOESM2_ESM.docx]

| Name | Sequence |
| --- | --- |
| RBCK1 Forward | 5‘ —GCAGATGAACTGCAAGGAGTATCA—3’ |
| RBCK1 Reverse | 5‘ —TGCAGCATCACCTTCAGCAT—3’ |
| 36B4 Forword | 5‘ —GGCGACCTGGAAGTCCAACT—3’ |
| 36B4 Reverse | 5‘ —CCATCAGCACCACAGCCTTC—3’ |
| CDKN1A Forward | 5‘ —GTGGCTCTGATTGGCTTTCTG—3’ |
| CDKN1A Reverse | 5‘ —CTGAAAACAGGCAGCCCAAG—3’ |
| BTG2 Forward | 5‘ —AGACGAGGCAAAGCGGTAAA—3’ |
| BTG2 Reverse | 5‘ —TCCAACCATTCACGGTCAGA—3’ |
| P53INP1 Forward | 5‘ —TATGCTGCCCCATTTCATTT—3’ |
| P53INP1 Reverse | 5‘ —CTGTGCATAACTCCTGCCCT—3’ |
| P53 Forward | 5‘ —TGCAATAGGTGTGTGCGTCAGAA—3’ |
| P53 Reverse | 5‘ —CCCCGGGACAAAGCAAA—3’ |
| siRBCK1 #1 | GGTGCACCTTCATCAACAA |
| siRBCK1 #2  siP53 | GGATTACCAGCGATTTCTA  AAGCGAGCACTGTCCAACAAC |

| His-P53 Protein Sequence |
| --- |
| MGSSHHHHHHSSGLVPRGSHMEEPQSDPSVEPPLSQETFSDLWKLLPENNVLSPLPSQAMDDLMLSPDDIEQWFTEDPGPDEAPRMPEAAPRVAPAPAAPTPAAPAPAPSWPLSSSVPSQKTYQGSYGFRLGFLHSGTAKSVTCTYSPALNKMFCQLAKTCPVQLWVDSTPPPGTRVRAMAIYKQSQHMTEVVRRCPHHERCSDSDGLAPPQHLIRVEGNLRVEYLDDRNTFRHSVVVPYEPPEVGSDCTTIHYNYMCNSSCMGGMNRRPILTIITLEDSSGNLLGRNSFEVHVCACPGRDRRTEEENLRKKGEPHHELPPGSTKRALSNNTSSSPQPKKKPLDGEYFTLQIRGRERFEMFRELNEALELKDAQAGKEPGGSRAHSSHLKSKKGQSTSRHKKLMFKTEGPDSD |
| GST-RBCK1 Protein Sequence |
| MSPILGYWKIKGLVQPTRLLLEYLEEKYEEHLYERDEGDKWRNKKFELGLEFPNLPYYIDGDVKLTQSMAIIRYIADKHNMLGGCPKERAEISMLEGAVLDIRYGVSRIAYSKDFETLKVDFLSKLPEMLKMFEDRLCHKTYLNGDHVTHPDFMLYDALDVVLYMDPMCLDAFPKLVCFKKRIEAIPQIDKYLKSSKYIAWPLQGWQATFGGGDHPPKSDLEVLFQGPLMDEKTKKAEEMALSLTRAVAGGDEQVAMKCAIWLAEQRVPLSVQLKPEVSPTQDIRLWVSVEDAQMHTVTIWLTVRPDMTVASLKDMVFLDYGFPPVLQQWVIGQRLARDQETLHSHGVRQNGDSAYLYLLSARNTSLNPQELQRERQLRMLEDLGFKDLTLQPRGPLEPGPPKPGVPQEPGRGQPDAVPEPPPVGWQCPGCTFINKPTRPGCEMCCRARPEAYQVPASYQPDEEERARLAGEEEALRQYQQRKQQQQEGNYLQHVQLDQRSLVLNTEPAECPVCYSVLAPGEAVVLRECLHTFCRECLQGTIRNSQEAEVSCPFIDNTYSCSGKLLEREIKALLTPEDYQRFLDLGISIAENRSAFSYHCKTPDCKGWCFFEDDVNEFTCPVCFHVNCLLCKAIHEQMNCKEYQEDLALRAQNDVAARQTTEMLKVMLQQGEAMRCPQCQIVVQKKDGCDWIRCTVCHTEICWVTKGPRWGPGGPGDTSGGCRCRVNGIPCHPSCQNCH |
