# Supplementary material for: RBCK1 promotes p53 degradation via ubiquitination in renal cell carcinoma
Source: Cell Death Dis. 2019 Mar 15;10(4):254. doi: 10.1038/s41419-019-1488-2 (PMC6420644; doi:10.1038/s41419-019-1488-2)
Supplement: Supplementary file 3 — Supplemental Figure legends [file 41419_2019_1488_MOESM3_ESM.docx]

**Supplementary Figure 1**

(A) Quantitation of relative glucose level in Caki-1 cells. siControl was compared to siRBCK1 #1 group/siRBCK1 #2 group separately. Error bars represent the mean ±SD of 3 independent experiments. *P<.05

(B) Quantitation of relative glucose level in 769-P cells. siControl was compared to siRBCK1 #1 group/siRBCK1 #2 group separately. Error bars represent the mean ±SD of 3 independent experiments. *P<.05
